# Risk of Recurrence of Symptomatic Intracranial Atherosclerosis in Posterior Circulation Seen to Be Higher Than That in Anterior Circulation in Long-Term Follow-Up

**DOI:** 10.3389/fneur.2020.574926

**Published:** 2020-11-06

**Authors:** Jingyu Zhang, Kai Zhang, Baixue Jia, Zhongqi Qi, Dapeng Mo, Ning Ma, Feng Gao, Zhongrong Miao

**Affiliations:** Interventional Neuroradiology Center, Beijing Tiantan Hospital, Capital Medical University, Beijing, China

**Keywords:** symptomatic intracranial atherosclerosis (sICAS), posterior circulation, recurrent stroke/TIA, medication, long-term outcomes

## Abstract

**Background:** Intracranial atherosclerotic stenosis (ICAS) is an important cause of ischemic stroke. In Asians, intracranial atherosclerotic disease leads to 33–50% of ischemic events. At present, treatment with medication vs. endovascular therapy (EVT) for symptomatic ICAS (sICAS) patients is still debatable. The clinical prognosis of patients who are not completely free of stroke symptoms despite regular medication and are not eligible for EVT for various reasons, is not yet investigated.

**Aim:** To report the long-term recurrence rate of stroke in a cohort of symptomatic ICAS patients who intended to undergo EVT upon admission but could not for various reasons after digital subtraction angiography (DSA) evaluation.

**Method:** This is a retrospective analysis of consecutive sICAS patients in a single center from January 1, 2016 to August 31, 2017 who underwent DSA assessment alone and were not eligible for further EVT. Demographic information, risk factors related to cerebrovascular disease, clinical comorbidities, medication, imaging data, and long-term outcomes were reported.

**Results:** A total of 218 patients were included in the study; 42 (19.2%) patients had recurrence of stroke/transient ischemic attack (TIA) at the 1-year follow up. Patients were divided into two groups according to lesions in anterior circulation (*n* = 120) or posterior circulation (*n* = 98). There was a higher stroke/TIA recurrence rate in the posterior circulation than anterior circulation group (25.5 vs. 14.2%, *p* = 0.035). Given the advanced age, higher prevalence of coronary heart disease, larger stenosis length, and poorer collateral circulation, the posterior circulation group showed a higher risk of recurrent stroke/TIA and death than the anterior circulation group [HR = 3.092, 95% CI (1.335–7.164), *p* = 0.0084], after adjusting for all confounding factors in the COX regression model. Kaplan–Meier analysis showed that sICAS recurrence and mortality risk in the posterior circulation group was consistently higher than that in the anterior circulation group (log-rank-test, *p* = 0.033).

**Conclusions:** Patients with posterior circulation sICAS have higher recurrence risk than those with anterior circulation managed with medication alone. Further, posterior circulation lesion is an independent risk factor for recurrence in sICAS patients.

## Introduction

Symptomatic intracranial atherosclerosis (sICAS) is one of the main causes of ischemic stroke in China and refers to the stenosis of one or more intracranial arteries with a stenosis rate of 50–99%, resulting in insufficient blood supply and transient or persistent neurological impairment, including transient ischemic attack (TIA) and ischemic stroke (1). In fact, the severity of vascular lesions in patients with ischemic cerebrovascular disease is not consistent with the clinical manifestations, as some patients still have a high risk of stroke recurrence after intensive medical treatment or endovascular therapy (EVT). In the Warfarin-Aspirin Symptomatic Intracranial Disease (WASID) trial, the 1-year recurrent rate of ischemic stroke for patients with a stenosis ≥50% was up to 15% (aspirin group) and 14% (warfarin group), and the respective 2-year recurrent rates were 20.4 and 17% (2). A prospective study of symptomatic atherothrombotic intracranial stenoses (GESICA) study indicated that the 2-year stroke recurrence rate of sICAS patients treated with drugs alone was 38.2% (3). In China, the prevalence of intracranial atherosclerotic stenosis (ICAS) was 46.6% (4), with a high risk of stroke recurrence. Nowadays, the treatment for patients with sICAS comprises medication and EVT, pre-dominantly including balloon dilatation and stent implantation. However, the published outcomes of two vital trials—SAMMPRIS (5) and VISSIT (6)—that compared the efficacy and safety between medication and EVT highlighted an overwhelming preference for medication in clinical practice (7). Recently, the lower peri-procedural complication rate reported in the Wingspan Stent System Post-market Surveillance (WEAVE) trial seemed to rectify the benefit of EVT for sICAS patients (8). The guidelines recommended medication as the primary treatment for sICAS patients, then asked neurointerventionalists to select “proper” patients for further EVT as an alternative, such as those with severe stenosis, hypoperfusion, or poor collateral circulation and those in whom medication was ineffective (9). Considering that more individualized treatment is required for sICAS patients, neurointerventionalists are urged to identify the true prognosis and recurrence risk of sICAS, especially in those who are not completely free of stroke symptoms despite regular medication and who do not qualify for EVT either.

Therefore, the aim of our study is to report the long-term recurrent rate of stroke in a cohort of sICAS patients who intended to undergo EVT upon admission but could not for various reasons after digital subtraction angiography (DSA) evaluation. We mainly compared the different characteristics and prognosis between sICAS patients with lesions in the anterior and posterior circulation, as this is still poorly investigated.

## Materials and Methods

### Study Design and Patients

**Figure 1:**
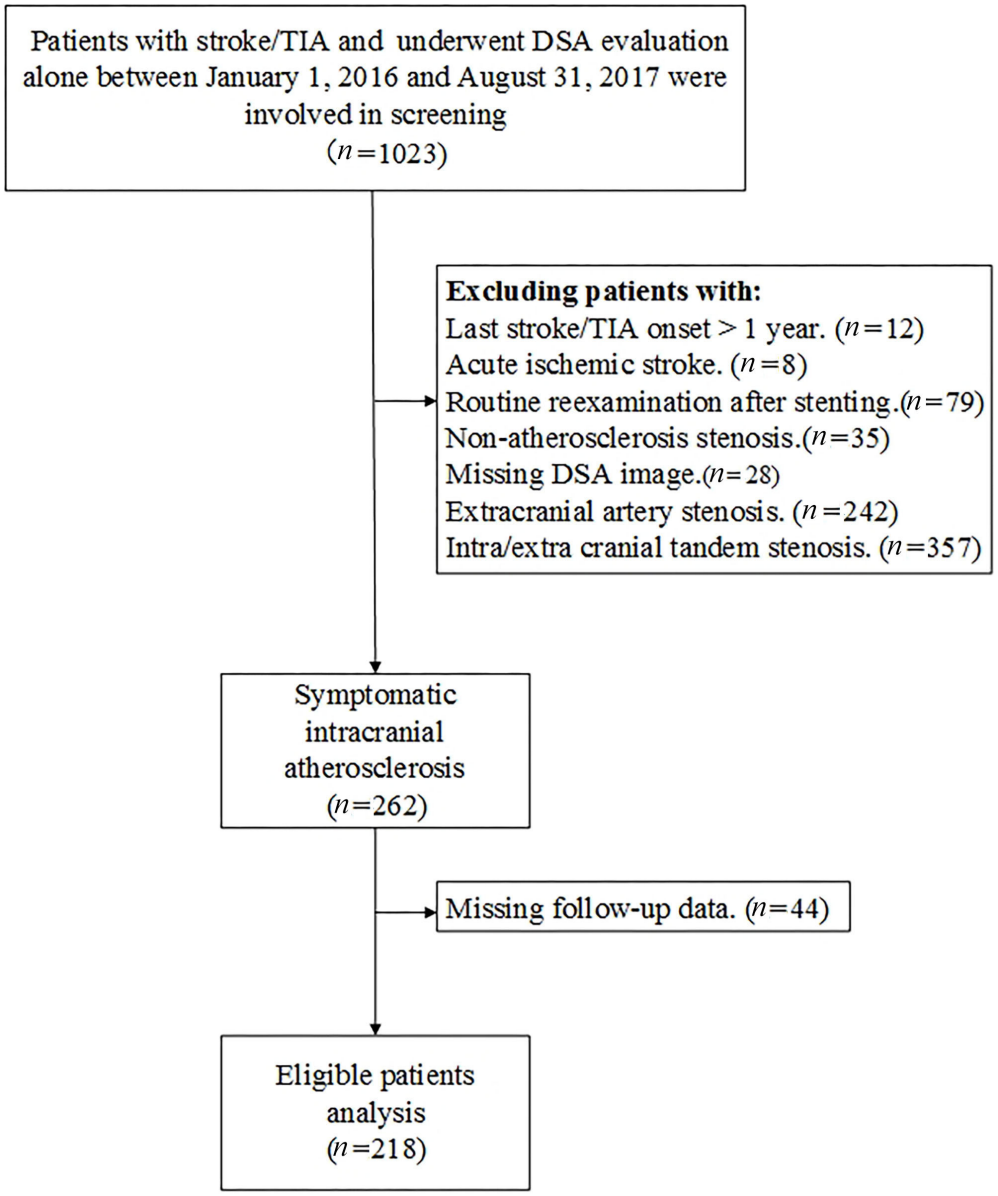
Flow chart of patient screening for the study.

This is a single-center, retrospective analysis of consecutive patients with sICAS who underwent DSA evaluation alone between January 1, 2016 and August 31, 2017. Demographic data, clinical characteristics, and imaging data were available in a prospectively developed local database. Patients with acute ischemic stroke, routine reexamination after cerebrovascular stenting, and concomitant extracranial artery stenosis and non-atherosclerosis stenosis were excluded. Those who underwent EVT within 1 year without new stroke/TIA onset after enrollment were also exclude from our analysis. A flow chart of patient screening is presented in Figure 1. Missing data were obtained from the patients' medical records. The study was approved by the institutional review board of the institution. All patients enrolled in the study signed informed consent for the procedure.

### Procedure

Because most patients aim to seek further EVT upon admission, the DSA procedure was focused on evaluating the feasibility and peri-procedural complication rate based on magnetic resonance angiography (MRA) and/or computed tomography angiography (CTA). At the same time, most patients had undergone intracranial CT perfusion (CTP) examination and had confirmed perfusion defects in the responsible lesions. Some patients also underwent high-resolution MRI examination of intracranial vessels to determine the existence of plaque and wall of responsible vessels.

All DSA procedures followed the standard guidelines and were performed by experienced interventionalists. The DSA evaluation parameters focused on the responsible lesions causing vascular stenosis and included lesion location, lesion length, degree of stenosis, angulation, calcification, eccentric plaques, antegrade blood flow, and collateral circulation in the ischemic area.

EVT was considered for patients with the following: (1) angiographically measured degree of ICAS ≥70%, which was associated with ischemic stroke or TIA; (2) distal hypoperfusion with an TICI (Thrombolysis in Cerebral Ischemia Scale) score of 0-2a; and (3) poor collaterals with an American Society of Interventional and Therapeutic Neuroradiology/Society of Interventional Radiology (ASTIN/SIR) Collateral Flow Grading System score of <3.

**Figure 2:**
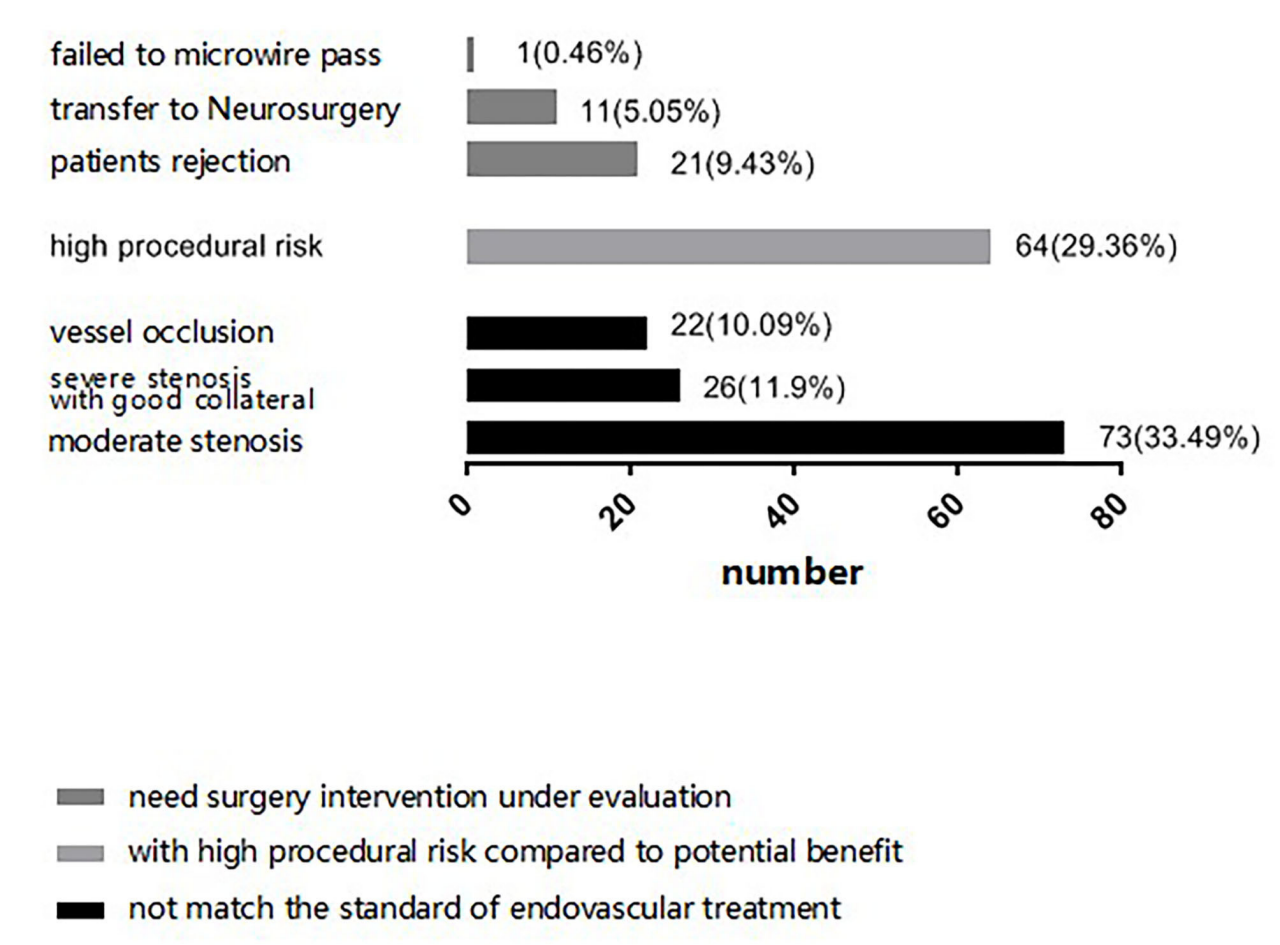
Various reason of failing to further endovascular treatment.

When select proper sICAS patients to EVT, surgical indication, technical feasibility, and risk of peri-procedural complications are the main concern for neurointerventionalists. In our center, we thought that stenosis with severe atherosclerotic plaque, complete occlusion, severe tortuous artery, stenosis lesion longer than 10 cm, multiple stenosis and abundant perforating vessels near the lesion vessels, bleeding tendency or severe coagulation dysfunction would increase the risk of peri-procedural complications. We first judge whether a sICAS patient has an indication of EVT, if yes, then assess technical feasibility, risk, and potential benefit. However, there were several patients rejected further EVT for economic distress although they were recommended to EVT by neurointerventionalists. And some patients were transferred to neurosurgery clinic because of failing to microwire pass or the condition of the stenosis distal vascular bed is poor. The final decision regarding further EVT depended on neurointerventionalists' evaluation and recommendation, as well as patients' and their family's preferences. The three situations in which patients were not eligible to receive further EVT are detailed in Figure 2.

### Peri-Procedural Medications

All patients took at least one kind of antiplatelet drug, including aspirin (100 mg/day) and/or clopidogrel (75 mg/day) for more than 1 week after the onset of ischemic stroke or TIA, and other long-term oral drug therapy for individual complications. At discharge, all patients were re-recommended to adjust the antiplatelet drug dose according to Essen Stroke Risk Score (ESRS) or ABCD2 score and thromboelastogram (TEG) results, for long-term secondary prevention. The type and dose of statins were selected according to individual conditions and lipoprotein metabolism level. It was recommended that the target value of blood pressure be <140/90 mmHg, the target value of glycosylated hemoglobin (HbA1C) be lower than 7%, the target value of low-density lipoprotein cholesterol (LDL-C) be lower than 1.8 mmol/L. At the same time, we also reminded patients regarding health management which included quitting smoking, losing weight, and performing appropriate physical activity.

### Follow-Up

Patients were followed-up at 3 months, 6 months, and 1 year after the procedure by means of clinic and telephone visits by the interventionalist. In the outpatient clinic, the doctor asked the patient whether there were any new ischemic events during the visit period, then asked in detail the symptoms, duration and relief of neurological deficit. The patients took CTA and/or MRA for cerebrovascular assessment. For those patients relieved within 24 h who complaint with same symptom as initial ischemic onset or related to the symptomatic stenotic artery, we concluded it a recurrent TIA. We defined stroke as an outcome event mainly on the basis of symptom and imaging of patients. When the patient's symptom got worse with NIHSS added 4 points, or the patient had new symptoms, and there were indeed new lesions confirmed by imaging, we thought the patient had an outcome event. If a patient was suspected of having a stroke during the telephone interview, his or her imaging would be traced and collected. If the imaging was done in our center, it would be reviewed by interventionalists to be confirmed. If the imaging was done in other hospital, the patient would be required to provide the imaging or its report. All the images of patients with suspected stroke were available.

The medication, management of critical risk factors, and new clinical complications and treatment, any type of adverse events during the follow-up period were also recorded. The final follow-up was carried out in October 2018.

### Imaging Review

All patients' DSA images were selected separately and reviewed by two interventionalists blinded to the patient details. Lesion location, length, degree of stenosis, TICI, and ASTIN/SIR grade were the main parameters. Stenosis was measured according to the standard of WASID. The consistency of both groups of measurement results was tested, and the results were reinterpreted by a third interventionalist in case of controversial interpretations.

### Statistics

Baseline characteristics are presented as means, medians, or percentages based on types of variables. Univariate associations between demographic, clinical, procedural variables, and the 1-year recurrent stroke were evaluated. COX regression model was used for multivariate analysis, and the recurrence of stroke in anterior and posterior circulation was evaluated by Kaplan–Meier curve. Stepwise logistic regression analysis was used to assess the relationship between the factors and 1-year recurrent stroke in different groups. A multivariate model was constructed using variables with *p*-values < 0.1 in the univariate analysis by forward inclusion. The *p*-value for inclusion in the model was <0.05. All analyses were done in IBM SPSS V.24 (International Business Machines Corp).

## Results

A total of 218 patients were included in the analysis and were divided into two groups: anterior circulation sICAS (*n* = 120) and posterior circulation sICAS (*n* = 98). The degree of stenosis and type of ischemic type showed no significant intergroup differences. Compared with the anterior circulation, Patients in the posterior circulation sICAS group were older (60.5 ± 9.1 vs. 56.1 ± 9.5, *p* = 0.0007); had greater prevalence of coronary heart disease (26.5 vs. 12.5%, *p* = 0.0084); larger stenosis length (7.6 ± 6.1 mm vs. 5.66 ± 3.43 mm, *p* = 0.0080); less AcoA opened (28.6 vs. 50%, *p* = 0.0013); and more PcoA opened (except for embryonic posterior cerebral artery, both unilateral and bilateral) (37.8 vs. 29.2%, *p* = 0.0137). In addition, posterior circulation sICAS showed poorer collateral circulation than the anterior circulation, as the numbers of ASTIN/SIR score of 0-2 and the TICI grade 0-2a were increased to 90.8 vs. 80.8% (*p* = 0.0380) and 57.1 vs. 41.7% (*p* = 0.0230), respectively (Table 1).

**Table 1:** Baseline characteristics.

**Characteristic**	**Anterior circulation (*n* = 120)**	**Posterior circulation (*n* = 98)**	***p***
Age (y)	56.1 ± 9.5	60.5 ± 9.1	0.0007
Male (*n*, %)	84 (70.0)	77 (78.6)	0.1520
BMI	25.7 ± 2.9	26.2 ± 2.8	0.2196
Hypertension (*n*, %)	85 (70.8)	77 (78.6)	0.1933
Hypercholesterolemia (*n*, %)	64 (53.3)	57 (58.2)	0.4750
Diabetes mellitus (*n*, %)	49 (41.0)	38 (38.8)	0.7580
CAD (*n*, %)	15 (12.5)	26 (26.5)	0.0084
Smoker (*n*, %)	66 (55.0)	55 (56.7)	0.8019
INR	0.97 ± 0.07	1.02 ± 0.28	0.0519
TEG-AA	89.51 ± 21.18	94.01 ± 14.83	0.1530
TEG-ADP	50.75 ± 24.73	55.45 ± 25.18	0.2540
Ischemic type			0.8922
TIA (*n*, %)	54 (45.0)	45 (45.9)	
Stroke (*n*, %)	66 (55.0)	53 (54.1)	
Onset to DSA procedure (*d*)	52 (30–94)	45 (26–96)	0.6371
Lesion location (*n*, %)			
ICA	42 (35.0)		
MCA M1	73 (60.8)		
MCA M2	5 (4.2)		
VA		39 (39.8)	
BA		59 (60.2)	
Stenosis rate	66.5 ± 20.9	69.5 ± 21.0	0.2930
Stenosis rank			0.2110
Mild (*n*, %)	28 (23.3)	12 (12.2)	
Median (*n*, %)	44 (36.7)	43 (43.9)	
Severe (*n*, %)	27 (22.5)	24 (24.5)	
Occlusion (*n*, %)	21 (17.5)	19 (19.4)	
Stenosis length	5.66 ± 3.43	7.60 ± 6.10	0.0080
AcoA	60 (50.0)	28 (28.6)	0.0013
PcoA (unilateral of bilateral)	35 (29.2)	37 (37.8)	0.0137
Complete Willis Circle	9 (7.5)	5 (5.1)	0.4735
Leptomeningeal collateral	99 (83.2)	69 (70.4)	0.0250
ASTIN 0-2 (*n*, %)	97 (80.8)	89 (90.8)	0.0380
TICI 0-2a (*n*, %)	50 (41.7)	56 (57.1)	0.0230

*Data are median (IQR) or n (%). BMI, Body Mass Index; CAD, Coronary Artery Disease; INR, International standard ratio; TEG-AA, Thromboelastogram platelet mapping arachidonic acid inhibition; TEG-ADP, Thromboelastogram platelet mapping adenosine diphosphate inhibition; TIA, Transient Ischemic Attack; DSA, Digital Subtraction Angiography; ICA, internal carotid artery; MCA M1, Middle Cerebral Artery M1 segment; MCA M2, Middle Cerebral Artery M2 segment; VA, Vertebral Artery; BA, Basilar Artery; AcoA, Anterior Communicating Artery; PcoA, Posterior Communicating Artery; TICI, Thrombolysis in Cerebral Ischemia Scale; ASTIN/SIR, American Society of Interventional and Therapeutic Neuroradiology/Society of Interventional Radiology Collateral Flow Grading System*.

We analyzed the reasons for not EVT between the two groups. There were 73 (60.8%) patients in anterior circulation group and 48 (49.0%) patients in posterior circulation group showed no indication for EVT, 28 (23.3%) patients in anterior circulation group and 36 (36.7%) patients in posterior circulation group with high procedural risk compared to penitential benefit, 19 (15.8%) patients in anterior circulation group and 14 (14.3%) patients in posterior circulation group were recommended EVT but they rejected or transferred to neurosurgery for treatment. There was no statistical difference between the two groups for not EVT (χ^2^ = 1.049, *p* = 0.306).

We recorded the medication and lifestyle management at each visit, partially referred to patients' self-reported blood pressure and blood glucose levels. Almost everyone took at least one type of antiplatelet drug at the 1-year follow-up. Patients with posterior circulation sICAS took aspirin for a longer time [12 months (IQR: 12–12) vs. 12 months (IQR: 3–12), *p* = 0.0121] and had a lower chance of stopping the antiplatelet drug (4.08 vs. 15.13%, *p* = 0.0073) than those with anterior circulation. Lifestyle management showed no differences between the two groups and were not satisfactory either (Table 2).

**Table 2:** Medication and lifestyle control at the 1-year follow-up.

**Measures**	**Anterior circulation (*n* = 120)**	**Posterior circulation (*n* = 98)**	***p***
Antiplatelet drugs (*n*, %)	108 (90.8)	95 (98.0)	0.0560
Time of aspirin (*m*)	12 (3–12)	12 (12–12)	0.0121
Time of clopidogrel (*m*)	12 (2.5–12)	6 (3–12)	0.1628
Discontinuation of antiplatelet (*n*, %)	18 (15.13)	4 (4.08)	0.0073
Discontinuation of statins (*n*, %)	26 (22.03)	17 (17.40)	0.3904
BP control (*n*, %)^a^	58 (68.24)	54 (70.13)	0.5240
Blood sugar control (*n*, %)^b^	18 (36.7)	14 (36.8)	0.8174
Quit smoking (*n*, %)	24 (45.28)	22 (45.83)	0.9558

a *Recommended target value of blood pressure be <140/90 mmHg*.

b *Target value of glycosylated hemoglobin (HbA1C) be lower than 7%*.

Overall, 42 (19.2%, eight stroke and 34 TIA) patients suffered stroke/TIA recurrence and five (2.29%) patients died of other non-ischemic cerebrovascular diseases by the 1-year follow-up. The posterior circulation group showed a higher stroke/TIA recurrence rate than the anterior circulation group (25.5 vs. 14.2%, *p* = 0.035). However, the type of ischemic event and recurrent-enrolment time showed no difference between the two groups. Further, majority had an independent functional prognosis (mRS ≤ 2) in both groups (Table 3).

**Table 3:** Comparison of the 1-year outcome in both groups.

**Endpoint events**	**Anterior circulation (*n* = 120)**	**Posterior circulation (*n* = 98)**	***p***
New territorial ischemic event	17 (14.2)	25 (25.5)	0.035
TIA	13 (76.5)	21 (84.0)	0.542
Stroke	4 (23.5)	4 (16.0)	
Time of recurrence to onset (*d*)	312 (164.5–393.5)	277 (81–397.5)	0.412
Independent outcome (mRS ≤ 2)	116 (99.2)	91 (94.8)	0.056
New ischemic cardiovascular events	2 (1.7)	4 (4.1)	0.278
Any hemorrhagic disease	7 (5.8)	10 (10.2)	0.231
Death	4 (3.3)	1 (1.02)	0.256

**Figure 3:**
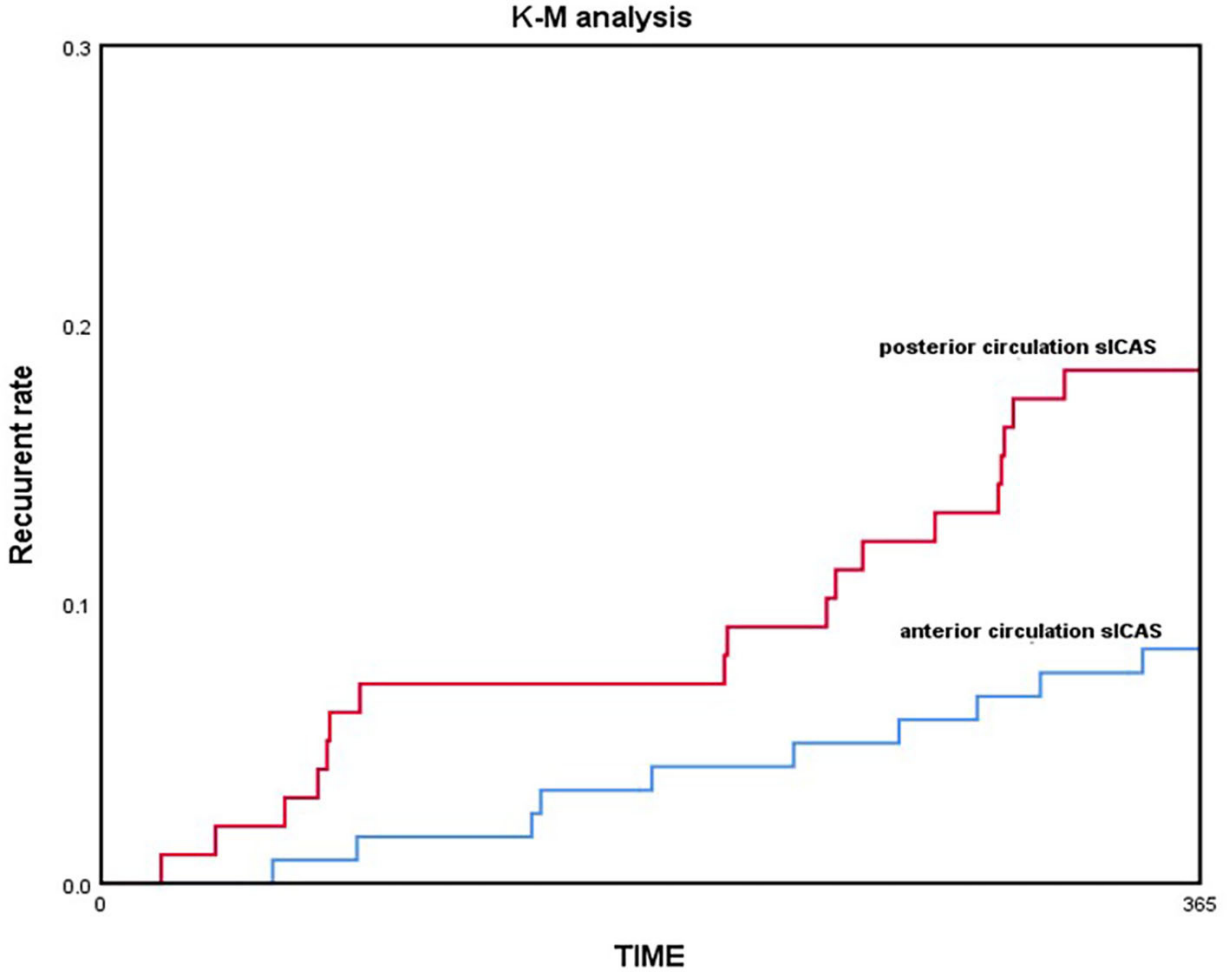
The 1-year recurrent rate compared between two groups using the Kaplan-Meier method.

COX regression model was used for multivariate analysis; the posterior circulation group still showed a higher risk of recurrent stroke/TIA and death than the anterior circulation group without any adjustment [HR = 1.932, 95% CI (1.043–3.578), *p* = 0.0362]; by adjusting only age and sex [HR = 2.221, 95% CI (1.170–4.217), *p* = 0.0147]; and by adjusting for all confounding factors [HR = 3.092, 95% CI (1.335–7.164), *p* = 0.0084; Table 4]. Kaplan–Meier analysis showed that the posterior circulation sICAS recurrence and mortality risk were consistently higher than the anterior circulation group in the long-term (log-rank-test, *p* = 0.033; Figure 3).

**Table 4:** Different HR values in survival analysis.

	**HR**	**95% CI**	***p***
Unadjusted	1.932	1.043–3.578	0.0362
Only age and sex adjusted	2.221	1.170–4.217	0.0147
All confounding factors adjusted*	3.092	1.335–7.164	0.0084

* *Contained age, sex, CAD, stenosis length, stenosis rate, TICI, ASTIN/SIR, AcoA, and PcoA*.

**Figure 4:**
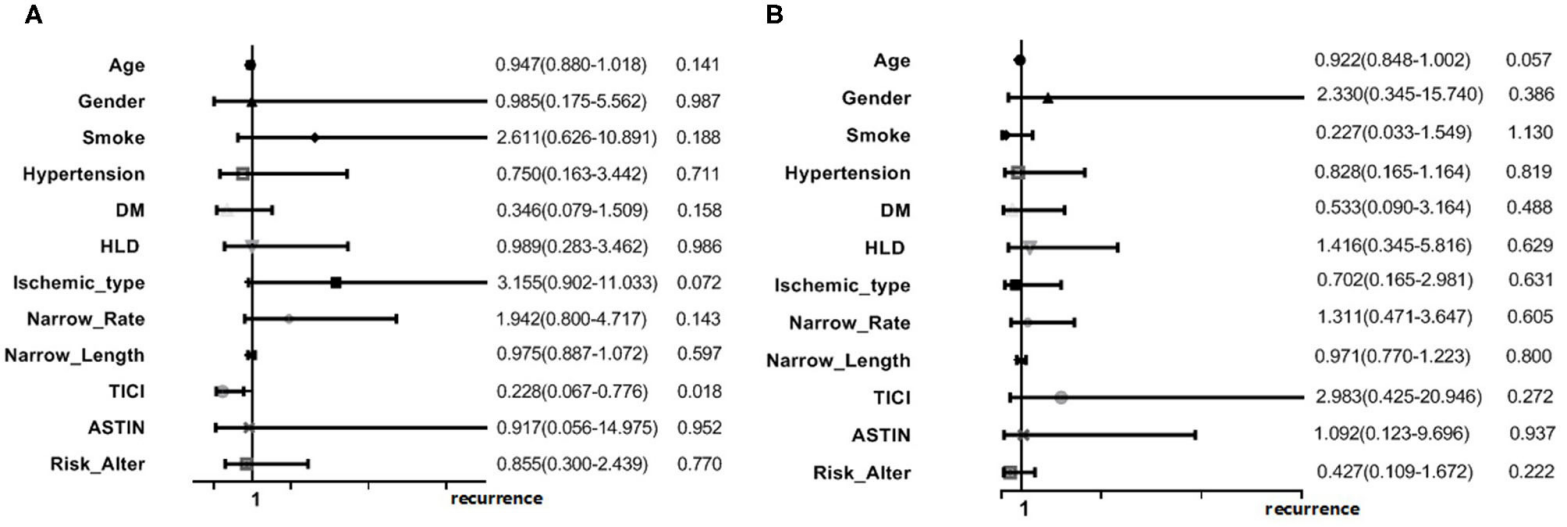
Multivariate analysis in posterior **(A)** and anterior **(B)** circulation sICAS.

Multivariate logistic regression analysis was performed in the two groups separately. In the posterior circulation sICAS group, good antegrade flow (TICI 3/4) could seemed to lower the recurrence risk [OR = 0.228 95% CI (0.067–0.776), *p* = 0.018]. No remarkable risk factors were found in the anterior circulation group (Figure 4).

## Discussion

Because of the considerable controversy with respect to optimum treatment for sICAS patients (medication vs. EVT), in this study, we evaluated a cohort of sICAS patients who intended to undergo EVT upon admission, but could not after DSA evaluation for various reasons. It is worthwhile to identify specific patients with different lesions, recurrent risk, and clinical outcomes for a precise therapy. Our study is a simple retrospective observational study, more like a description of the real state.

In our study, all patients received early standard medical drug therapy such as with antiplatelets and statins, and other risk factor intervention after initial ischemic event onset, and then sought EVT as a rescue treatment when regular medication did not help. Regardless of the exact reasons for not undergoing further EVT, these sICAS patients who did not benefit from medical management may benefit from EVT (10, 11).

Consistent with previous studies, most patients in our study were male (73.9%), and majority showed common risk factors of ischemic stroke, which was consistent with the characteristics of intracranial atherosclerosis. However, there were a few differences. First, the median time of onset to enrollment was 51 days (IQR: 28–94), which is significantly longer than the 30-day time window of the classic SAMMPRIS and VISSIT study and the 7-day time window of the CICAS study. Second, majority lesions caused mild-to-moderate (74.56 ± 20.43%) stenosis that could be the potential sICAS population worthy of intensive treatment. According to CISS classification (12), the pathogenesis of ischemic stroke was classified from high to low as parent artery-perforator lesion in 47 cases (39.5%), low perfusion in 44 cases (37.5%), artery-to-artery embolism in 17 cases (14.3%), and mixed mechanism in 11 cases (9.2%), which is slightly different from previous studies: low perfusion accounted for 52.4% in the SAMMPRIS and artery-to-artery embolism accounted for 50.7% in the WASID trials.

Ischemic recurrence was observed in 42 (19.2%) patients at the 1-year follow-up in our study, higher than that in the medical arms of the SAMMPRIS (12.2%) and VISSIT (15.1%) trials. This may be because more than half of the patients (64.7%) in our study were complicated with multiple ICAS, and several studies indicated that the increased burden of ICAS was an independent risk factor for recurrent stroke (13–15). Another potential reason is that the proportion of patients with posterior circulation sICAS was 45% in our study, which was also higher than that reported in previous studies. Patients with posterior circulation sICAS showed more ischemic recurrence than those with anterior circulation (25.5 vs. 14.2%), which has not been proven in previous studies. Despite the many differences between the two groups at baseline, posterior circulation sICAS were found to be independent risk factors for stroke recurrence in patients with sICAS in the COX survival analysis. In addition, the effect of early drug treatment was not good in our study. Although antiplatelet drugs and statins were prescribed and risk factor intervention was emphasized upon after admission, there were no Aggressive Medical Measures like in the SAMMPRIS trial in real life (16), which has been proved in follow-up, medication and lifestyle control is not qualified as requested. Based on a large sample of Chinese ICAS patients, the CICAS registration study pointed out that the risk of stroke recurrence increases with the increase of the number of risk factors (4).

Since sICAS patients with unsatisfactory treatment have a high risk of recurrent ischemic events, it is more clinically meaningful to identify specific populations with a high risk of recurrence. A subgroup analysis based on the WASID study (17) showed that the 1-year stroke recurrence occurred more frequently in ICAS patients with >70% stenosis and within 17 days from onset to enrollment. Different lesion locations and subtypes of ischemic events did not seem to have an influence. However, previous studies preferred to choose patients with anterior circulation sICAS, because the diverse clinical symptoms caused by posterior circulation are challenging to determine.

In our study, we found that posterior circulation sICAS patients are older and more commonly have coronary atherosclerosis disease than those with anterior circulation. According to the DSA results, lesion length is significantly larger in posterior circulation and collateral circulation is poorer than when compared to AcoA/PcoA existence and TICI/ASTIN score. Maybe the limit of sample size and hospital bias, no more different characteristics were found as former study. Without doubt, there are differences between anterior and posterior circulation sICAS (18, 19), not only in demographic characteristics and pathogenesis of ischemic stroke but also in risk of recurrence. In our study, the comparison of primary outcome showed that the recurrence rate is higher in the posterior circulation group than the anterior circulation group (25.5 vs. 14.2%, *p* = 0.035), but there was no difference in the type of ischemic events. It was seen that the recurrence risk (including 1-year recurrent ischemic events and death) in the posterior circulation sICAS group was higher than that in the anterior circulation group throughout, by gradually adjusting the confounding factors in the COX survival analysis. Kaplan–Meier analysis also indicated that recurrence was more likely in posterior circulation sICAS than anterior circulation sICAS with increasing time. This points toward the need for a larger prospective trial that will exclusively focus on the prognosis and treatment of posterior circulation sICAS.

We tried to assess the potential factors related to the recurrent ischemic events in the posterior circulation sICAS patients by multiple analysis. The logistic regression model showed good antegrade flow (TICI 3/4) could lower the recurrence risk in posterior circulation sICAS, [OR = 0.228, 95% CI (0.067–0.776), *p* = 0.018]. Hypoperfusion is most likely a feature connected with recurrent stroke. The time from onset to DSA was 45 days (IQR: 26–96) in the posterior circulation group, and the collateral circulation was also poor. Regardless of whether the cerebral blood flow reserve and regulation differ in anterior and posterior circulation stroke, this is a bold hypothesis. Maybe a limit of traditional collateral circulation measurements which are applied for anterior circulation stroke more properly.

Former studies also showed other risk factors in patients with posterior circulation sICAS. A stroke registration study on a Taiwanese population pointed that the increased degree of vertebrobasilar artery (VBA) stenosis was associated with stroke recurrence. The 1-year stroke recurrence risk of patients with moderate-to-severe VBA stenosis was 1.21-times higher than that of patients with mild VBA stenosis [95% CI (1.01–1.45), *p* < 0.05] (20). Patients with basilar artery stenosis had a higher risk of recurrent stroke than those with vertebral artery stenosis according to a meta-analysis (21). Another retrospective study of a small sample pointed out that most cases of posterior circulation stroke were caused by parent artery-perforator lesion, in which case the likelihood of recurrence was the least (22).

The main limitation of our study is the retrospective nature and small sample size. Moreover, patient enrollment bias and department bias caused by hospital cannot be eliminated. We only selected patients who underwent DSA evaluation in our department for 20 months. In fact, most patients missed screening because of the difficulty of continuous follow-up. Another limitation is the collection of follow-up information. Most recurrent ischemic events in our study are TIA-related, with the majority being repeat TIA attacks since the initial onset. After enrollment, although medication did not greatly resolve patient symptoms, risk-factor intervention was not feasible. Maybe we should have compared sICAS patients treated with EVT in the same period to determine whether recovery of blood flow has any effect on recurrence. A further study is on-going to clarify the prognosis of sICAS and precise therapy.

## Conclusion

Patients with posterior circulation sICAS have higher recurrence risk than those with anterior circulation on medication alone, and posterior circulation lesion is an independent risk factor for recurrence in sICAS patients. Further studies should be performed exclusively on patients with posterior circulation sICAS.

## Data Availability Statement

The raw data supporting the conclusions of this article will be made available by the authors, without undue reservation.

## Ethics Statement

The studies involving human participants were reviewed and approved by Ethics committee of Beijing Tiantan Hospital. The patients/participants provided their written informed consent to participate in this study.

## Author Contributions

ZM designed and supervised the study. JZ and KZ was responsible for data analyses, drafting, and revising the article. BJ and ZQ was responsible for data acquisition. DM, NM, and FG were involved in revising the article for important intellectual content. All authors critically reviewed the article and approved the final version.

## Conflict of Interest

The authors declare that the research was conducted in the absence of any commercial or financial relationships that could be construed as a potential conflict of interest.
